# Bin it or pin it? Which professional ethical guidance on managing COVID-19 should I follow?

**DOI:** 10.1186/s12910-020-00491-5

**Published:** 2020-07-15

**Authors:** Richard Huxtable

**Affiliations:** Centre for Ethics in Medicine, Population Health Sciences, Bristol Medical School, Canynge Hall, 39 Whatley Road, Bristol, BS8 2PS UK

**Keywords:** COVID-19, Coronavirus, Ethical guidance, Professional guidance

## Abstract

**Background:**

As the COVID-19 (coronavirus) pandemic develops, healthcare professionals are looking for support with, and guidance to inform, the difficult decisions they face. In the (current) absence of an authoritative national steer in England, professional bodies and local organisations have been developing and disseminating their own ethical guidance. Questions inevitably arise, some of which are particularly pressing during the pandemic, as events are unfolding quickly and the field is becoming crowded. My central question here is: which professional ethical guidance should the professional follow?

**Main body:**

Adopting a working definition of “professional ethical guidance”, I offer three domains for a healthcare professional to consider, and some associated questions to ask, when determining whether – in relation to any guidance document – they should “bin it or pin it”. First, the professional should consider *the source of the guidance*: is the issuing body authoritative or, if not, at least sufficiently influential that its guidance should be followed? Second, the professional should consider *the applicability of the guidance*, ascertaining whether the guidance is available and, if so, whether it is pertinent. Pertinence has various dimensions, including whether the guidance applies to this professional, this patient and/or this setting, whether it is up-to-date, and whether the guidance addresses the situation the professional is facing. Third, the professional should consider *the methodology and methods by which the guidance was produced*. Although the substantive quality of the guidance is important, so too are the methods by which it was produced. Here, the professional should ask whether the guidance is sufficiently inclusive – in terms of who has prepared it and who contributed to its development – and whether it was rigorously developed, and thus utilised appropriate processes, principles and evidence.

**Conclusion:**

Asking and answering such questions may be challenging, particularly during a pandemic. Furthermore, guidance will not do all the work: professionals will still need to exercise their judgment in deciding what is best in the individual case, whether or not this concerns COVID-19. But such judgments can and should be informed (and constrained) by guidance, and hopefully these preliminary observations will provide some useful pointers for time-pressed professionals.

## Background

“Guidelines for medical management are now part of medical life. A fool – loosely defined as someone who does not know much about a particular area of medicine – will do well to follow guidelines when treating patients, but a wise man (again, loosely defined as someone who does know about the disease in question) might do better not to follow them slavishly” [1].

As the COVID-19 (coronavirus) pandemic develops, even “wise” healthcare professionals are – understandably – looking for support with, and guidance to inform, the difficult decisions they face. In the UK, as elsewhere, there has been extensive recent activity, nationally, regionally and locally, but – until 1 April 2020 – national *ethical* guidance was conspicuous by its absence. April then saw the publication of two key documents offering professional ethical guidance related to COVID-19. The Royal College of Physicians (RCP) led the way [2], with the support of numerous other bodies, followed shortly thereafter by the British Medical Association (BMA) [3].

Hopefully such guidance will prove useful to clinicians on the front-line and will help to promote the interests of not only individual patients, but also the wider population. However, questions will also arise. Who should have the responsibility – and authority – to issue such guidance? What is the status and import of this guidance? Are the different guidance documents consistent – and should they be? And if guidance does conflict, which (if any) should take priority?

These sorts of questions had been brought sharply into focus for me during an ongoing (but, necessarily, interrupted) research project that explores treatment decisions for incapacitated patients, as my collaborators and I were struck by the plethora of guidance for health and social care professionals working for and with such individuals [4]. This is far from the only context in which (actual or purported) guidance proliferates; others might be reminded of pre- (and presumably post-) COVID-19 efforts to issue guidance pertaining to artificial intelligence (AI), including its deployment in healthcare.

The issue returns now, with considerable force, as various organisations are publishing or preparing guidance on ethical patient care during COVID-19. Informal discussions with colleagues indicate that everyone appreciates the need for such guidance, but most are keen to avoid unnecessary duplication, whilst also recognising that discrete guidance might be needed for different patient groups, professionals, settings and contexts. So how should the presumed audiences of the guidance – such as healthcare professionals – navigate this cluttered terrain? In the following, my focus is on professional ethical guidance in the UK, which I will attempt to define momentarily, and specifically on guidance pertaining to medical (or healthcare) ethics; although the COVID-19 pandemic has provided the spur, many of the observations will apply beyond this specific context.

## Bin it or pin it?

### What is professional ethical guidance?

First, it is worth briefly reflecting on what counts as professional ethical guidance. Medical ethics (or, more broadly, healthcare ethics or, even more broadly, bioethics) is an applied, multi- and inter-disciplinary field. Some argue that the field is fundamentally concerned with issuing “practical oughts” i.e. making *convincing* claims that actually serve to *convince* the relevant stakeholders ([5], p. 57). Medical ethics therefore aspires to make a difference in the “real world” and, on this sort of account, everything issued in the name of “medical ethics” can be considered “guidance” in a sense.

But “medical ethics” is a broad field, populated and produced by diverse actors. *Professional ethical guidance* occupies a distinct sector of the field. Taking each word individually, this: (1) is aimed at (particular) professionals and (typically) prepared and issued by professional organisations; (2) is ethical insofar as it articulates what *should* be done or which attitudes *should* be cultivated; and (3) aspires to guide the clinical practice of the professionals to whom it is addressed.

Sometimes, as with the recent RCP and BMA documents, professional ethical guidance will be explicitly labelled as such. But even guidance that does not explicitly talk of “ethics” is likely implicitly to rest on – or advance – particular value commitments or presuppositions. However, failure to engage explicitly with ethics might mean that, however inadvertently, important values are missed. For example, the first version of NICE’s rapid guidelines on critical care during COVID-19 made no direct reference to ethics [6]. Unfortunately, the guidelines appeared to invite judgments against some individuals with long-term disabilities that were considered unjustly discriminatory; following legal challenge, the guidelines were re-drafted, although the second version also omits any direct reference to ethics [7, 8].

Here, however, I essentially focus on the former i.e. professional guidance that *is* explicitly articulated as ethical in nature and import. It is nevertheless worth acknowledging that the identification of “ethical” guidance will not always be straightforward. The potential for confusion can be further compounded by the variety of terms used to describe such documents – what (if any) are the differences between guidance, guidelines, protocols, pathways, Codes of Practice, and the like? Whatever the relevant document might be called, however, my central question here is: which guidance (etc.) should the professional follow?

### Which professional ethical guidance should I follow?

There are mixed practical, prudential and ethical reasons for ensuring that healthcare professionals know which guidance they should follow. Knowing which guidance to follow will, for example, help to ensure that professionals are *clear* about they are expected to do and how to do it, *consistent* in their practices, and that those practices are *defensible*, because they take due account of pertinent facts and values, including ethical values.

But how should a professional determine which *specific* guidance is for them – and, by extension, their patients or service users? There appear to be various factors for a professional to consider when determining whether this or that guidance is the one for them i.e. whether the professional should, as Norman put it in a 2013 blog, “consign it to the bin, or to pin it to the notice board with fairy lights around it” [9].

Norman cites a 2012 ruling, which examined local guidance concerning the mobility of the visually impaired, in which Kenneth Parker J said:

“In my view, the weight that should be given to particular guidance depends upon the specific context in which the guidance has been produced. In particular (without intending to create an exhaustive list) I believe that it is necessary to give due regard to the authorship of the guidance, the quality and intensity of the work done in the production of the guidance, the extent to which the (possibly competing) interests of those who are likely to be affected by the guidance have been recognised and weighed, the importance of any more general public policy that the guidance has sought to promote, and the express terms of the guidance itself” ([10], para 39.).

Whilst not exhaustive, these observations strike in the right direction. Drawing on them, the following offers an attempt to map some of the pertinent factors and questions to ask, on which I hope others will be inclined to build. The factors are organised into three domains: (1) the source of the guidance; (2) the applicability of the guidance; and (3) the methodology and methods by which the guidance was produced.

#### The source of the guidance

First, the professional should consider *the source of the guidance* i.e. the provenance of the guidance and thus the authors to whom Kenneth Parker J referred. A key question to ask here is:

- How authoritative is the source of the guidance?

Given its provenance, some guidance will be authoritative and, indeed, non-negotiable and binding. Legal guidance – deriving from primary legal sources, like Acts of Parliament or judges’ rulings, and secondary sources, like Regulations or Codes of Practice – will typically fall into this category. So too will (“quasi-legal”) guidance that is issued by the professional’s regulator, which occupies what Miola calls the “formal” sector of ethical discourse ([11], p. 6).

Guidance from, for example, the General Medical Council (GMC) will therefore be authoritative for (and over) doctors. As the BMA explains,

“The GMC decide which doctors are able and qualified to work in the UK, set the standards that doctors must follow throughout their career, and take action when those standards have not been met” [12].

Indeed, guidance issued by the GMC has sometimes been cited with approval in, and has correspondingly informed, the law [13]. Failure to heed the guidance (indeed, edicts) of the law or the regulator would not be prudent, since this may lead to loss of liberty or livelihood. The same might be said of guidance that is issued by employers. As such, one way of discerning the authority of guidance involves identifying the possible ramifications of failing to comply.

Turning specifically to COVID-19, professionals should be guided by the pronouncements and principles emanating from the law and professional regulators. Some of these will be well-established and should already be familiar to professionals, although the pandemic has also prompted some revisions to the law in England and Wales [14]. As yet, however, no authoritative position has been taken in England and Wales on some of the thornier ethical questions that COVID-19 might pose, for example, about how medical resources such as ventilators in ICU should be allocated, should the need to make such decisions arise.

COVID-19 raises a myriad of such questions, including about patients who do *not* have the virus, but who still need treatment, care and support, and whose needs should not be entirely clouded by the “COVID fog”, as a clinical colleague has so vividly expressed it [15]. Ideally there would be ethical guidance from an authoritative source, such as the Government, through the Department of Health, Chief Medical Officer (CMO) or similar authority [15–19]. Scotland has issued such guidance, which clearly conveys the pertinent ethical principles and signals the need for clinical ethics support during the pandemic [20]. That document does not offer detailed practical protocols, but it does offer a useful framework, although its publication has been rather overshadowed by events concerning the (then) Scottish CMO [21].

England and Wales currently lack such a statement, so professional, regional and local organisations are stepping in to fill the gap. Notwithstanding the merits of the guidance that is emerging here (and, indeed, internationally), the COVID ethics field is becoming crowded [22], but the available guidance lacks the overt authority of the Scottish statement. Where this is the case, the professional might next ask:

- How influential is the source of the guidance?

Although they fall short of having binding authority, other sources will still be highly influential, meaning their guidance is likely to be worth heeding.^1^ This may be particularly true of those organisations that have influence over any authoritative position that is adopted; where this is the case, prudence might again be a motivator.

Miola has referred to a “semi-formal” sector of ethical discourse, occupied by the BMA and Royal Colleges [11]. The BMA is not only “the largest registered trade union” for UK doctors, but also works.

“with governments to lobby for improvements to health care. Also a professional body, the BMA leads debate on ethical, scientific and public health matters through research and publications” [12].

The BMA may be the largest union, but it is not the only one, which necessarily implies that there will be non-members to whom its guidance might appear superfluous. Even BMA members might wonder whether they should or must always heed its guidance. But both members and non-members would be advised to recognise the esteem in which the BMA, and its ethical guidance, is held and to appreciate its influence over professional medical practice. As the above statement suggests, the BMA has the ear of Government and “leads debate”. In doing so, as some judges have noted, the BMA will of course seek to heed the law and convey important legal messages to its audience [23]. But sometimes the influence will extend in the opposite direction, with BMA guidance *informing* the authoritative legal position, such as when a court approves or adopts particular guidance as reflective of the legal position [24].

Similar observations may be made about the Royal Colleges. As the BMA explains, these.

“are professional membership bodies that you can join … dependent on your area of practise. They support fellows and members throughout their career, work to advance standards and improve patient care, and develop policy and guidance” [12].

Membership might be required at some career stages – for example, when undergoing specialist training – but not every professional will be or will remain a member of the relevant College. However, like the BMA, the Royal Colleges can also “inform” the authoritative position ([25], para. 12).

Even organisations that have less overt influence over any *authoritative* position that is adopted might still have influence in practice. The Nuffield Council on Bioethics, for example, is “an independent body that informs policy and public debate about the ethical questions raised by biological and medical research”, but it has no official status as such [26]. Despite this, I understand from clinical colleagues that the detailed guidance it issues on a range of topics has been used to inform clinical practice.

As such, even where the source is not strictly authoritative, what it says may have a bearing on any authoritative position, or otherwise prove influential in shaping professional practice.

#### The applicability of the guidance

Second, the professional should consider *the applicability of the guidance* by looking, as Kenneth Parker J suggests, to “the express terms of the guidance itself”. In short, the guidance should be capable of doing what it sets out to do i.e. guiding professional practice.

A first key question to ask is:

- Is the guidance accessible?

This is self-evident and need not be laboured: if guidance is meant to guide, those who are to be guided need to be able to locate it.^2^ Fortunately, the internet can help with accessibility but, even so, the guidance will need to be clearly signposted to those who need it and not hidden away. However, open access and clear signposting is unlikely to be sufficient. As COVID-19 unfolds, the digital landscape threatens to become congested, as various bodies – national, regional and local – publish their guidance. Ideally, as noted above, there would be national leadership, offering a uniform clear message, which would minimise the risks of inconsistency, confusion or (simply) unnecessary searching by professionals who will be under considerable strain. In the absence of an authoritative national steer, bodies drafting guidance would be advised to co-ordinate and streamline their efforts as best they can.

Whether or not the guidance results from a national conversation, once the professional has obtained it, she or he will need to be able to act on it. Here a second key question arises:

- Is the guidance pertinent?

There are various dimensions to the question of pertinence, raising (at least) five further questions to ask and answer. First, is the guidance meant to apply to this professional? Obviously, guidance directed at doctors – or specific groups thereof – might not be pertinent to others.

Second, does the guidance apply to the particular patient or group of patients in question? Whilst some ethical norms and principles will be applicable to different people with different conditions, guidance dealing with, for example, children and young people will not – without further work – be equipped to inform the professional practice of geriatricians.

Third, does the guidance apply in the relevant setting – clinical and/or geographical? During COVID-19, there is likely to be guidance targeted specifically towards intensive care settings, but alternative guidance is likely to be needed for those working in other settings (such as home care). Equally, there may be jurisdictional or other geographical considerations: guidance for Wales, Scotland and Northern Ireland might not be applicable in England, and so too guidance directed at, for example, urban locales might have less pertinence in a rural setting.

Fourth, is the guidance up to date? Temporality matters: some guidance might well stand the test of time – note, for example, how emerging COVID-19 guidance builds on principles first articulated in relation to the 2009 flu pandemic [28] – but other guidance will, if it is not updated or is superseded, fall into redundancy or desuetude. As COVID-19 poignantly demonstrates, the world can move on quickly, meaning guidance might need to be revised to reflect changing circumstances.

Finally, and linked to the last point, does the guidance in question address the situation the professional is facing? Sometimes a document will offer guiding principles, which might prove helpful in the absence of any more specific pointers. However, on other occasions, the guidance might fail significantly or entirely to point the professional towards an answer to their particular question or dilemma.

In summary, no matter how authoritative or influential the issuing body, if the purported guidance is inaccessible or not pertinent, it will not be capable of application, in which case professionals will need to look elsewhere for the answers they seek.

#### The methodology and methods by which the guidance was produced

The professional should next consider *the methodology and methods* which led to the production of the guidance.^3^ This may be challenging, particularly when time is scarce, as is likely to be the case during a pandemic. The task may be harder still if the guidance is insufficiently transparent about the processes that led to its creation. However, where possible, as Kenneth Parker J puts it, account should be taken of “the quality and intensity of the work done”.

The “quality of the work” can be judged in different ways. The *substantive* quality or legitimacy of the guidance that is produced will be a consideration. Whether the guidance gets it “right” – and how this might be judged – are undoubtedly important questions. For example, COVID-19 threatens to raise (inter alia) thorny questions of resource allocation and distributive justice, and concerns have already been raised about the defensibility of the principles and positions offered in some national guidance [29]. However, here (returning to Kenneth Parker J’s phrasing) I will focus on the quality “of the work done” to produce the guidance. The quality of the process cannot guarantee the quality of the product, but it might at least offer an indication in this direction. A key question to ask here is:

- Is the guidance appropriately inclusive?

Guidance should be appropriately inclusive, in terms of who prepares it and who contributes to its development. First, the group that prepares the guidance should reflect the range of experiences, perspectives and expertise that will be needed to address the topic at hand.^4^ The Nuffield Council on Bioethics indicates how it seeks to capture the relevant range of voices:

“For each in-depth inquiry that we undertake, we convene a multi-disciplinary working group. We appoint a Chair and work with them to appoint working group members from a range of disciplines (for example, science, law, theology, philosophy, industry)” [30].

Second, in developing its position, the group should ensure it listens to the relevant stakeholders. As Kenneth Parker J says, guidance can be judged by “the extent to which the (possibly competing) interests of those who are likely to be affected by the guidance have been recognised and weighed”. Those affected by the guidance should have their voices heard and heeded, perhaps as members of the issuing group, but certainly as contributors to its work. Returning to the Nuffield Council, its in-depth inquiries strive to hear from a range of people with experience, via meetings or written submissions [30]. As recent events have shown, failure to heed the maxim “Nothing about us without us!” will understandably provoke criticism and may necessitate revision [29, 31].

Even if not every stakeholder is able to help draft or inform the guidance, care will still be needed to ensure that their opinions and interests are accounted for. This leads on to further considerations of evidence and process, captured by a second overarching question:

- Was the guidance rigorously developed?

Rigour, too, has different dimensions. Amongst the questions to ask are: has the guidance been developed through an appropriate process? The processes might vary by organisation, but some sort of process will be needed – as will transparency about what this involves. NICE, for example, is legally required “to have, and consult on, procedures for giving advice or guidance, and making recommendations”, which it conveys via detailed process and methods manuals [32].

Next, what are the principles which informed the development of the guidance? Although sadly not always the case, sometimes the principles that underpin the development and, ultimately, the content of the guidance will be openly articulated. NICE guidelines, for example, are informed by a (publicly available) set of principles, which were updated in January 2020 to replace its previous “values framework” [32]. Of course, NICE is not explicitly aiming to issue *ethical* guidance, although (as it appears to appreciate) its work will implicitly adopt ethical positions. As for those who *are* engaged in preparing overtly ethical guidance, they will sometimes make their principled commitments explicit too. The RCP’s COVID-19 guidance, for example, explains up-front that “The principal values that inform this guidance are that any guidance should be accountable, inclusive, transparent, reasonable and responsive” [2]. Each value is then outlined in the document, as well as in a column published by the Chair [33]. Such statements can help the reader, including in their judgment as to the quality of the resulting guidance.^5^

Leaving that aside, it is worth asking what evidence has been gathered to inform the guidance? Guidance is likely only to be as good as the evidence that has contributed to its development. Good ethics requires good facts [35], and this can be challenging, as we have seen in the early days of COIVD-19, when robust evidence is not yet available. Despite the challenges, many organisations appreciate the importance of good evidence. The Nuffield Council, for example, states that “Gathering evidence is a major part of all of our projects. We thoroughly research each topic and consider a wide range of views,” and they outline the various ways in which they seek to gather evidence [30]. Similarly, NICE notes that its “guidance and standards are underpinned by evidence. So we need to ensure that this evidence is relevant, reliable and robust” [32].

Of course, even robust evidence needs to be used robustly. Finally, then, one may ask how evidence has been used to inform the guidance – in particular, how has the evidence been assessed and analysed? Different sorts of evidence might help to answer different sorts of questions; the quality of evidence will vary; limitations should be recognised; and interpretations should be clear and careful [1].

## The fool, the wise person, and the need for both judgment and guidance

More can and should be said about the range of factors to consider, and questions to ask, of professional ethical guidance. Although I hope there is something of value in these remarks, what I offer here is a necessary but not yet a fully sufficient blueprint or, indeed, a fully worked-up guidance *to* guidance.

But even if we can craft such a blueprint, more will always be needed. However authoritative, applicable and robust a piece of guidance may be, it will rarely provide the whole answer. According to Hampton:

“Medical treatment by a wise man who depends on an assessment of each individual patient and the application of expertise, judgement and common sense, will usually be preferable to a slavish application of guidelines” ([1], p. 28).

Whether the professional in question is the “fool” or the “wise person” that Hampton envisages, professional judgment will always be needed, including when applying guidance for professionals.

However, this observation invites an objection: if professionals will always be required to exercise their judgment, then is there any need to have guidance or, more specifically, to worry about which guidance (if any) they should “pin or bin”? The interplay between judgment and guidance is complex and merits more attention than I can devote here. However, some preliminary remarks can be offered about the need for both elements and about their (symbiotic) interaction.

### The need for judgment

Starting with the need for judgment, Coles observes that this is a feature of the professional role:

“Professionals are asked to engage in complex and unpredictable tasks on society’s behalf, and in doing so must exercise their discretion, making judgments – decid[ing] what is ‘best’ in the particular situation rather than what is ‘right’ in some absolute sense” [36].

Even guidance that seeks to stipulate the “right” behaviour will tend to leave room for – indeed, require – professional judgment as to what is “best” in the situation at hand. Guidance might not provide for every eventuality, meaning there will be situations where the professional will need to judge what is “best”. But such judgments are also likely to be needed, even when the available guidance is comprehensive, because the professional is likely to be required to discern how “best” to translate the principles into action in their particular situation. For example, (authoritative) guidance from the GMC sets out the principles governing consent to treatment, which notes (inter alia) that “How much information you share with patients will vary, depending on their individual circumstances”. ([37], para. 7) This obviously requires a judgment to be made by the professional in the individual case. In short, however much guidance seeks to furnish the (“right”) answer, more work is likely to be needed in order to determine what is “best”.

Some will nevertheless object to the need for judgment and the lack of “answers”. Medical ethics sometimes comes under fire in this regard. Many of us working in the field will have encountered the complaint that, despite a plethora of principles, processes, and the like, medical ethics does not provide professionals with “the answer”, as if this is a fair charge and a distinctive failing of the field. The charge is not wholly fair: there are areas in which medical ethics *has* converged on answers, such as about the aforementioned importance of obtaining consent from a patient or research participant.^6^ The charge is also not peculiar to medical ethics: judgment is also needed in other fields, including others which purport to guide human behaviour. Law, for example, will sometimes provide clear answers, but – particularly in an adversarial legal system – there will sometimes be different views as to what “the law” instructs, which barristers will fight out in court, before a judge then – literally – makes the relevant considered judgment.

We might even be disquieted if all the “answers” *were* to be provided in advance, whether by ethics, law, or professional guidance. Coles’ reference to the “best” and the “right” hints at a distinction between the *particular* and the *general*. A focus on the general, in the form of off-the-peg “answers”, might, at worst, tend towards absolutism and thus towards a view of the “right”, which is imposed on everyone, regardless of whether it is indeed “right” or even “best” for every individual. Such a focus risks overlooking or overwhelming the particular, and thus the individual patient, context or decision.

This was effectively the basis of the challenge to the NICE guidance: general guidance had not given due regard to the rights and interests of those with particular disabilities [29]. Examples like this not only reinforce the need to ensure that guidance is appropriately informed by stakeholders, but also remind us that general propositions might need to be tailored to the specific person, situation or decision.

Tailoring obviously requires the exercise of judgment. As the Chair of NICE has pointed out, even evidence-based medicine needs professional discretion, “otherwise doctors would have been replaced by robots long ago” [38]. Of course, robots have in fact taken on some roles from doctors, albeit such robots are not (yet?) fully autonomous, so they do still require human control and oversight. Indeed, tragic recent events involving the surgical Da Vinci robot demonstrate that the rise of robotics has not removed the need for (human) judgment [39]. Other recent controversies – such as the perceived “checklist” approach that was taken to the Liverpool Care Pathway for caring for the dying – further emphasise the more general point that deciding what is “best” involves more than the thoughtless, mechanistic “application” of guidance or protocols [40].

### The need for guidance

Failing to exercise judgment as to what is “best” can therefore lead to harm. But this does not mean that the professional is free to pick and choose what they should do. Judgment is not formed and exercised in a vacuum, and guidance may play an important role in informing and constraining judgment.

Kaldjian defines clinical judgment as

“the basic skill of the physician that solves a medical problem through data collection, development and testing of explanatory hypotheses, and formulation of recommendations for therapy based on those hypotheses” [41].

Such judgment certainly requires the application of the professional’s knowledge, skills and experience – but it should also be informed by ethical values. Judgment appears to relate to the Aristotelean notion of “practical wisdom”, the virtue which enables us to use the right means to reach the right goals [41]. The relationship has been characterised in different ways. Pellegrino and Thomasma, for example, claim that clinical judgement *requires* practical wisdom: the ethical goals of medicine give clinical judgment its orientation [42]. Kaldjian, meanwhile, argues that “clinical judgement, if integrated with goals of care and ethical reasoning, is actually *a form of* practical wisdom within the specific context of medicine” ([41], p. 560, emphasis added).

I suggested above that knowing what guidance to follow will help to ensure that professionals are *clear* about they are expected to do and how to do it, *consistent* in their practices, and that those practices are *defensible*. These two accounts of the connection with practical wisdom shed further light on the question of defensibility, because they suggest that – in one way or another – clinical judgment is closely entwined with *ethical values*. Of course, professional ethical guidance will not necessarily capture the “right” ethical values; even guidance that has been issued by a recognised professional body, is applicable and has resulted from a rigorous process offers no guarantee that it is the “right” (ethical) guidance. Indeed, the view taken by a professional body might be judged to be out-of-step with (for example) the (internal) morality of the profession or with what individual professionals believe to be “right” [43]. But, if nothing else, professional ethical guidance may be the best available proxy for ensuring that professionals are alert to, and observant of, ethical values. This may explain why one professional organisation has described professional judgment as:

“Applying knowledge, skills and experience, in a way that is informed by professional standards, laws and ethical principles, to develop an opinion or decision about what should be done to best serve clients” [44].

As this account suggests, judgment should be informed – and, by extension, constrained – by (inter alia) professional guidance.

In sum, the relationship between judgment and professional ethical guidance is complex, and no doubt deserves further attention. For now, I hope to have indicated that there is a symbiotic relationship between the two: not only will judgment be needed to (for example) interpret and apply professional ethical guidance, but so too will guidance be needed to inform and constrain professional judgment.

## Conclusion

Guidance – however detailed and robust – can therefore inform judgment, but it cannot entirely replace it. Professionals will still be required to make hard ethical choices about what is best in the given case, which may leave a moral residue and, sadly, lead to moral distress [45]. At the same time, however, judgment can and should be aided by guidance, which should at least offer some moral comfort and support. But here our central question returns: how should a professional determine which *specific* guidance is for them i.e. when should they “pin it or bin it”? Although the relationship between judgment and guidance merits further exploration, my main aim here has been to begin a discussion about the factors that healthcare professionals should consider when deciding which professional ethical guidance to follow.

Guidance is valuable because it can clarify professional obligations and support consistent and defensible practice. Ideally, during a pandemic, those goals would be met through the provision of *authoritative* national ethical guidance for healthcare professionals, calls for which have been increasing in the UK [15–19].^7^ Of course, it is possible there might be more than one such document – and this is even more likely if there is no authoritative guidance, so other groups seek to fill the vacuum. Whether they are confronted with one guidance document or many, I have suggested that healthcare professionals should (at least, so far as the constraints imposed by the current pandemic permit) consider the provenance of the guidance, its applicability to their work, and the methodology and methods by which the guidance was produced. Hopefully, by considering these factors and asking a series of related questions, healthcare professionals can decide whether, in the case of the guidance before them, they should bin it or pin it.

## Data Availability

Not applicable.

## Abbreviations

AI: Artificial intelligence
BMA: British Medical Association
CMO: Chief Medical Officer
NICE: National Institute for Health and Care Excellence
RCP: Royal College of Physicians

## Footnotes

1 My focus here is on guidance issued by professional organisations, but I recognise that other groups or even individuals might also have influence, given (for example) the esteem in which they are held or the networks in and with which they work. Whatever the source of the guidance, authority and influence are not the only factors to consider: the other domains (below) offer further suggestions for assessing the soundness of the proposals issued.

2 This recalls one of Fuller’s conditions for the “Internal morality of law” – promulgation – which need to be observed if law is to fulfil its goal of guiding human behaviour via rules [27]. The other conditions are that legal rules should be general (as opposed to specific edicts), prospective, clear, non-contradictory, relatively constant, and not require the impossible, and that there should be congruence between the rules as stated and applied. Given their action-guiding orientation, these requirements might usefully be heeded by professional ethical guidance too, albeit adjusted to suit such guidance, rather than the dictates of law per se.

3 Whilst a discursive piece such as this one seldom includes an outline of method, in the spirit of what follows, I should acknowledge here the method by which I prepared this article. As the guidance landscape in the UK became increasingly cluttered and difficult to navigate, I discerned a need for “guidance” on selecting guidance. Clinical colleagues confirmed the growing need, so I elected to undertake a rapid narrative review, via Google (and Google Scholar) searches. In less time-pressed circumstances, a more robust approach would be followed, drawing on expert advice about the search strategy. The article should be read accordingly i.e. as a discursive piece, and acknowledging the rapidity and the subjectivity inherent in the method by which it was prepared.

4 As COVID-19 unfolds, there have been (understandable) concerns raised, not only about the credentials of some of the “experts” purporting to issue advice, but also about the voices that are not being heard, including those of people with disabilities [31]. Guidance that is appropriately inclusive is likely to inspire more confidence than guidance which is not. Of course, even the input and support of numerous stakeholders can offer no absolute guarantee as to the quality of any guidance that is produced. However, professionals might be more reassured by, and should likely heed, guidance which has been prepared – or at least endorsed – by a range of stakeholders, which takes due account of a range of pertinent perspectives and interests.

5 Judgment will also be required in the application of guidance, especially if the guidance is based on or articulates *multiple* substantive or procedural principles, because these will not always point in the same direction. This point will be very familiar to medical ethicists [34], and has rightly been recognised in some of the guidance being employed during the current pandemic, such as the (pre-COVID-19) pandemic flu framework [28]. I return to the question of judgment below.

6 Albeit in different ways, with different emphases and caveats.

7 As this article was being finalised, barristers at a leading chambers, 39 Essex Chambers, announced that they had been instructed “by two leading law firms to challenge the lack of a national framework for treatment prioritisation, if demand for life-sustaining treatment outstrips supply during the COVID-19 pandemic. Both claims are brought by people who are concerned that they will be deprioritised for treatment as a result of their impairments or additional health needs” [46]. Authoritative national guidance will surely follow if the case succeeds, but, although this would address the first of the domains presented here, the other domains would remain live concerns.
